# Abscess of the ligamentum teres hepatis and Arantius' ligament: A case report

**DOI:** 10.4314/ahs.v25i1.5

**Published:** 2025-03

**Authors:** Xiaopeng Suo, Jianfei Chen, Shuying Wang, Keming Zhang

**Affiliations:** 1 Department of Hepatobiliary surgery, Peking University International Hospital, Beijing 102206, China; 2 Department of General Surgery, Beijing Shijitan Hospital, Capital Medical University, Beijing, China; 3 Ministry of Science and Education, Peking University Eighth Clinical Medical College, Beijing 102206, China

**Keywords:** Ligamentum teres hepatis, Arantius' ligament, Abscess, Case report

## Abstract

Simultaneous abscesses of the ligamentum teres hepatis and Arantius' ligament are an extremely rare entity. The ligamentum teres hepatis (LTH) and the Arantius ligament are formed by the regression of the umbilical vein and venous ducts during embryonic period and are normally atretic in adulthood, which may recanalize in the case of portal hypertension. Even if the ligament is recanalized, in most cases only blood flows through it. It is very rare for abscesses to occur in these ligaments. Here we present a case with simultaneous LTH abscess and Arantius ligament abscess, a condition that has never been reported before. A 72-year-old female patient complained of epigastric discomfort and was diagnosed with an intra-abdominal tubular mass, which was confirmed to be the thickened LTH and Arantius ligament during operation. Postoperative specimens showed pus in the ducts and histopathological examination confirmed that the mass was the inflamed ligament. The patient's symptoms disappeared after the operation and there was no recurrence after 19 months of follow-up. Postoperative case data and related literature were reviewed. We here described in detail the characteristics and possible etiology of the disease.

## Introduction

Ligamentum teres hepatis (LTH) is formed from embryonic atresia of the left umbilical vein, communicating with the left hepatic vein or inferior vena cava through the ligamentum venosum. The ductus venosus is the continuation of the umbilical vein, which allows a large part of the oxygenated blood from the placenta to join the supradiaphragmatic inferior vena cava, bypassing the fetal liver and connecting directly to the right atrium. The ductus venosus is styled “Ductus Arantius” after Giulio Cesare Arantius who is thought to have discovered it^1^. Within one week of birth, the neonate's ductus venosus degenerates and is replaced by the ligamentum Arantius. LTH and ligamentum Arantius are attached to the capsule portion and the sagittal portion of the left branch of the portal vein, respectively. For clinicians encountering this condition for the first time, it can be very confusing. It is important for the clinician to be familiar with this disease. This article provides a detailed description of possible etiology, clinical manifestations, imaging features, and treatment options for this type of abscess to provide a reference for clinical decision making.

## Case presentation

**Figure 1:**
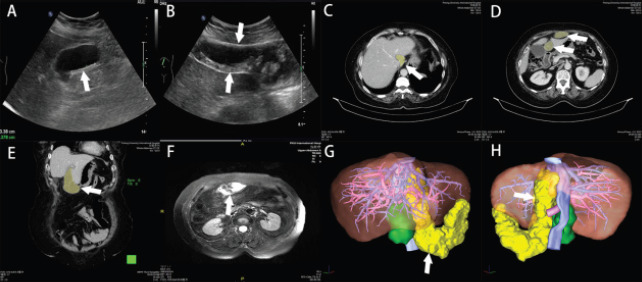
A panel of US, CT, MR, and Three-Dimensional reconstruction. (A) US showing sludge and sand particles in the gallbladder (arrow). (B) US showing a solid cystic mass on the abdominal wall (between the two arrows). (C) Axial CT view of the caudate lobe of the liver (arrow and yellow area). (D) Axial CT view of liver fissure zone and abdominal wall (arrows and yellow areas). (E) Coronal CT view of abscess of LTH (arrow and yellow area). (F) MR of a cystic mass with solid mass (arrow). (G) 3D reconstruction of the shape of LTH (arrow and yellow area). (H) 3D reconstruction of the ligament Arantius (arrow and yellow area)

A 72-year-old woman presented to the hospital with recurrent epigastric discomfort dating back 20 years, which had aggravated in the past 2 months. This patient had a history of calculous cholecystitis and was given intermittent conservative treatment. No history of periumbilical infection. The patient came to Peking university international hospital in March 2021. Laboratory examination showed an increase in CRP level (57.74 mg/L). The patient was negative for all of the neoplasm indicators tested (AFP, CEA, CA199, and CA724). An abdominal ultrasound (US) revealed a 9.3 × 6.1 × 3.4 cm mass with a clearly defined boundary and shape, (Figure 1A B). An abdominal CT scan during hospitalization revealed an abnormally dense lesion near the caudal lobe of the liver, crossing the second hilar region and the hepatic fissure, and connecting to the periaqueductal area of the anterior abdominal wall. (Figure 1C, D and E). An MRI of the upper abdomen further confirmed an irregularly shaped cystic mass showing high signal on both T1 and T2 and clear boundaries. It was observed near the anterior abdominal wall (Figure 1F). A 3D reconstruction was performed to further visualize the mass. The pack itself was colored yellow (Figure 1G and H).

**Figure 2:**
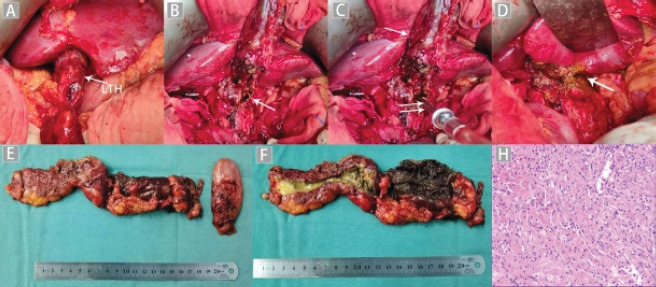
A panel of intraoperative photographs, postoperative specimen, and pathology. Intraoperative photographs: (A) The enlarged LTH (arrow). (B) The abscess extending into the liver (arrow). (C) Aspirator (double arrows) sucked up internal abscesses (double arrows). (D) After the complete removal of the ductus Arantius abscess and necrotic tissue (arrow). Postoperative specimen: (E) The resected LTH and gallbladder. (F) The specimen was dissected to reveal pus and necrotic tissue. Pathology: (G) A large infiltration of inflammatory cells

Considering the nature of the patient's symptoms and the failure of conservative treatment to provide complete relief, we performed an exploratory laparotomy. A sausage-like mass was seen extending from the hepatic ligament to the arantius duct. We resected the mass completely. Cholecystectomy was also performed under clinical indications. (Figure 2A–D). The operation lasted approximately 280 minutes. The patient recovered and was discharged from the hospital 9 days after surgery. The surgical specimen was about 20 cm long, and the dissection of the specimen revealed that it was filled with pus and necrotic tissue. Histopathological examination showed a large infiltration of inflammatory cells. At 19 months of follow-up, the patient had no further upper abdominal discomfort and no recurrence (Figure 2E–G).

## Discussion

Abscess of the LTH, a remnant of the obliterated fetal left umbilical vein, is rarely reported in the literature. Fujikawa et al.^2^ reported that only 18 cases of abscess formation of LTH had been identified in the English literature until 2020. To our knowledge, no abscess of the ligamentum venosum, also known as Arantius' ligament, a remnant of the ductus venous, has yet been reported in English. The simultaneous occurrence of LTH and Arantius ligament abscesses are much rarer.

In this case, both LTH and Arantius' ligament abscesses were present and contributing to the patient's symptoms. However, the etiology of the abscess of the ligaments teres hepatis and Arantius' ligament remains unclear. According to previous reports, abscess of LTH may be associated with acute calculous cholecystitis^3^, acute obstructive cseptic thrombosis^4^, pancreatitis^5^, and omphalitis^6^. Another study^6^ has reported two cases of a falciform ligament abscess secondary to an omphalitis. Contiguous spread of the infection via the round ligament may be its etiology.

In the current case, this patient also had cholecystitis. According to previous research^7^, the blood flows through chole-cystic veins directly into the intrahepatic portal branches^8^. This venous network might explain the extension mechanism of the abscess formation of LTH and ductus Arantius. We suspect that this patient might have had left-sided portal pyemia secondary to cholecystitis, spreading this infection to the LTH and ligament Arantius. This hypothesis has an anatomical foundation: the LTH and the ligament Arantius are connected to the left branch of the portal vein, which may be reopened to allow the passage of blood due to mutation or specific etiology under extreme pressure, such as portal hypertension or cirrhosis^9^. There was no evidence that the patient had portal hypertension or cirrhosis. We think the patency of her LTH and ductus Arantius may be due to incomplete latching after her birth.

Arakura et al.^4^ reported a similar case of abscess of the round ligament of the liver that was possibly caused by Acute obstructive suppurative cholangitis (AOSC) and septic thrombosis at the left portal vein. They speculate that portal thrombosis of the left branch played a major role in the formation of abscess in their case, and this path of infection may support the etiology hypothesis of our present case. Once it has formed, the abscess of the LTH may cause peritonitis, portal thrombosis, sepsis, and external compression leading to intestinal obstruction^10^. There is a report documenting that an abscess of the round ligament can extend to generalized peritonitis and necessitate emergency surgery^11^. Conservative treatment has been successful in some cases^4^,^10^. However, surgery is the main treatment for this kind of disease. In the current case, puncture drainage may not cure the lesion completely. This is because pus is sticky and difficult to remove by puncturing. Conservative treatment including puncturing is associated with relapse.

## Conclusion

In conclusion, abscesses of both the LTH and the ligament Arantius rarely present in the same patient. Such a condition can be diagnosed using imaging methods such as CT and treated with surgery with a relatively good prognosis. The etiology of the abscesses, however, should be investigated thoroughly for future reference. Theoretically, any disease that can cause portal vein pyemia, especially cholecystitis, choledocholitis, biliary pancreatitis, and umbilicitis, may cause abscesses in the LTH or ligament Arantius, because of the close anatomic relationshp between the ligament and the portal vein's left branch. The reopening of the involved ligaments serves as the leading risk factor for inflammation in such rare cases. We believe that this report will inspire clinicians to delve further into the problem to better understand the disease.
